# Clinical target volume (CTV) automatic delineation using deep learning network for cervical cancer radiotherapy: A study with external validation

**DOI:** 10.1002/acm2.14553

**Published:** 2024-10-14

**Authors:** Zhe Wu, Dong Wang, Cheng Xu, Shengxian Peng, Lihua Deng, Mujun Liu, Yi Wu

**Affiliations:** ^1^ Department of Digital Medicine School of Biomedical Engineering and Medical Imaging Army Medical University (Third Military Medical University) Chongqing China; ^2^ Yu‐Yue Pathology Research Center Jinfeng Laboratory Chongqing China; ^3^ Department of Radiation Oncology Zigong First People's Hospital Sichuan China; ^4^ Department of Radiotherapy Beijing Luhe Hospital Beijing China

**Keywords:** auto‐delineation, cervical cancer radiotherapy, clinical target volume, deep learning, generalization

## Abstract

**Purpose:**

To explore the accuracy and feasibility of a proposed deep learning (DL) algorithm for clinical target volume (CTV) delineation in cervical cancer radiotherapy and evaluate whether it can perform well in external cervical cancer and endometrial cancer cases for generalization validation.

**Methods:**

A total of 332 patients were enrolled in this study. A state‐of‐the‐art network called ResCANet, which added the cascade multi‐scale convolution in the skip connections to eliminate semantic differences between different feature layers based on ResNet‐UNet. The atrous spatial pyramid pooling in the deepest feature layer combined the semantic information of different receptive fields without losing information. A total of 236 cervical cancer cases were randomly grouped into 5‐fold cross‐training (*n* = 189) and validation (*n* = 47) cohorts. External validations were performed in a separate cohort of 54 cervical cancer and 42 endometrial cancer cases. The performances of the proposed network were evaluated by dice similarity coefficient (DSC), sensitivity (SEN), positive predictive value (PPV), 95% Hausdorff distance (95HD), and oncologist clinical score when comparing them with manual delineation in validation cohorts.

**Results:**

In internal validation cohorts, the mean DSC, SEN, PPV, 95HD for ResCANet achieved 74.8%, 81.5%, 73.5%, and 10.5 mm. In external independent validation cohorts, ResCANet achieved 73.4%, 72.9%, 75.3%, 12.5 mm for cervical cancer cases and 77.1%, 81.1%, 75.5%, 10.3 mm for endometrial cancer cases, respectively. The clinical assessment score showed that minor and no revisions (delineation time was shortened to within 30 min) accounted for about 85% of all cases in DL‐aided automatic delineation.

**Conclusions:**

We demonstrated the problem of model generalizability for DL‐based automatic delineation. The proposed network can improve the performance of automatic delineation for cervical cancer and shorten manual delineation time at no expense to quality. The network showed excellent clinical viability, which can also be even generalized for endometrial cancer with excellent performance.

## 1 INTRODUCTION

Cervical cancer is a serious and prevalent gynecology cancer, particularly threatening women's reproductive health. It is the fourth most common malignancy in females globally.^1^ The global incidence of cervical cancer is approximately 500 000 cases annually.^2^ Radiotherapy is a cornerstone of treatment in most stages of cervical cancer, and concurrent chemotherapy bestows additional survival benefits.^3^

Clinical target volume (CTV) delineation accurately is pivotal in ensuring the delivery of an enough and effective radiation dose to the tumor while sparing critical organs from radiation field damage. Usually, the CTV of radical cervical cancer consists of the uterus, cervix, para‐cervix, and upper 1/2 of the vagina, and the pelvic lymph nodes regions (such as internal iliac, obturator, external iliac, and common iliac lymph nodes). For post‐operated patients, the CTV is para‐cervix, vaginal stump, and pelvic lymph nodes regions. Accurate delineation of the CTV remains difficult due to the unclear boundaries of the gross tumor and sub‐clinical target regions and the individual variations are also big.^4^ Additionally, manual delineation is not only time‐consuming, and labor‐intensive but also prone to intra‐ and inter‐observer variations among oncologists with different experience levels.^4^ In recent years, deep learning (DL) has gained significant popularity in the semantic segmentation of medical images.^5^, ^6^, ^7^ Thus, it might be both reasonable and necessary to develop a quick and effective DL‐aided tool for CTV automatic delineation, which can reduce the workload of the oncologists and save medical resources.

It is challenging to delineate the CTV of cervical cancer automatically for both radical and post‐operated patients using DL because of the complexity of the targets. Only post‐operated patients^8^ or radical patients^9^ in specific tumor staging were studied in previous literature. Kim et al.^10^ evaluated the feasibility of automatic delineation of CTV for patients with endometrial and cervical cancer using atlas‐based auto‐segmentation (ABAS) algorithms, which are used in commercial software, making the research not available for many radiotherapy centers. DL‐based networks were open‐source, which were developed in many studies.^11^, ^12^, ^13^ In addition, the DL models of automatic delineation for cervical cancer studies did not have external validation,^10^, ^11^, ^12^, ^13^ and only a few researches had clinical evaluation.^9^ The generalization and robustness of the DL models are still unclear and their clinical applicability is also unknown. To deal with the above problems, more efforts should be devoted to constructing a model with external validation.

In this study, we proposed a novel DL network (ResCANet), which trained on one center's cervical cancer patients, and then validated the network via internal and external independent cervical cancer datasets. The clinical feasibility of CTV automatic delineation was evaluated both geometrically and subjectively. Furthermore, the delineation performance of the model was validated in endometrial cancer to explore the clinical applicability to another treatment site.

## 2 MATERIALS AND METHODS

### 2.1 Data collection and preparation

Data from 236 cervical cancer radiotherapy patients (39530 slices) from 2019 to 2022 at the Zigong First People's Hospital (ZG Hospital) were collected. Radical (*n* = 63) and post‐operated (*n* = 173) cervical cancer patients were both chosen. The planning CT images were obtained using a Brilliance CT Big Bore (Philips Healthcare, Best, Netherlands) according to a standard clinical acquisition protocol: 120 kVp, 120–300 mAs, 0.8 × 0.8 pixel spacing, 512 × 512 image size, and 3 mm slice thickness.

**FIGURE 1:**
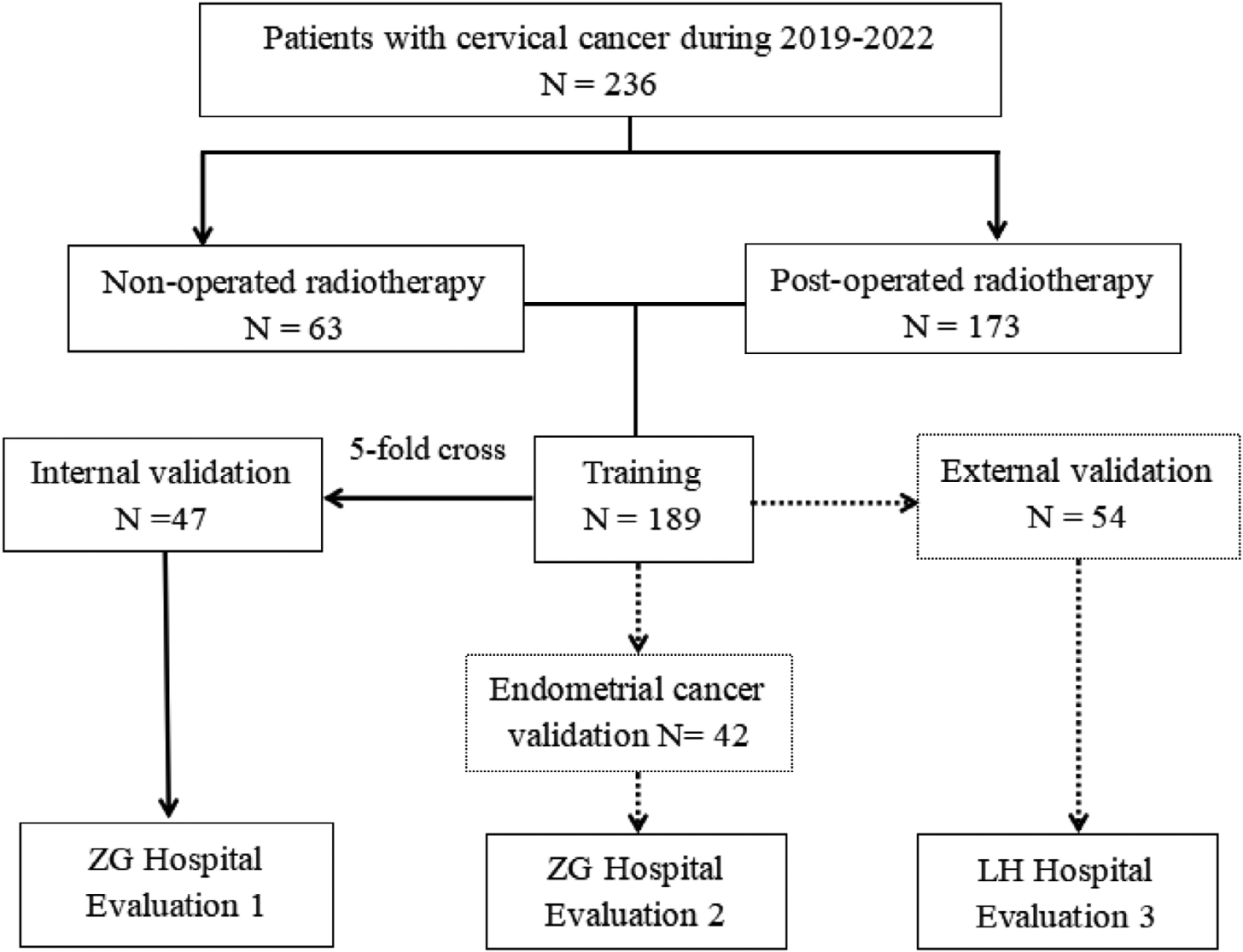
Details of the datasets.

Manual CTV delineation was finished in Eclipse 13.6 treatment planning system (TPS) (Varian Medical System Inc., USA) based on the guidelines of Radiation Therapy Oncology Group (RTOG). The delineation results were reviewed and approved by a senior oncologist (with more than 20 years of working experience). Data of 236 patients were randomly divided into 80% and 20% for 5‐fold cross‐validation, namely 189 patients for training and 47 patients for internal validation. In addition, a total of 54 patients (4610 slices, thickness = 5 mm) with cervical cancer from Beijing Luhe Hospital (LH Hospital) and 42 patients (6539 slices, thickness = 3 mm) with endometrial cancer from ZG Hospital were used as independent validation sets. Details of the datasets are presented in Figure 1.

### 2.2 DL model for CTV automatic delineation

**FIGURE 2:**
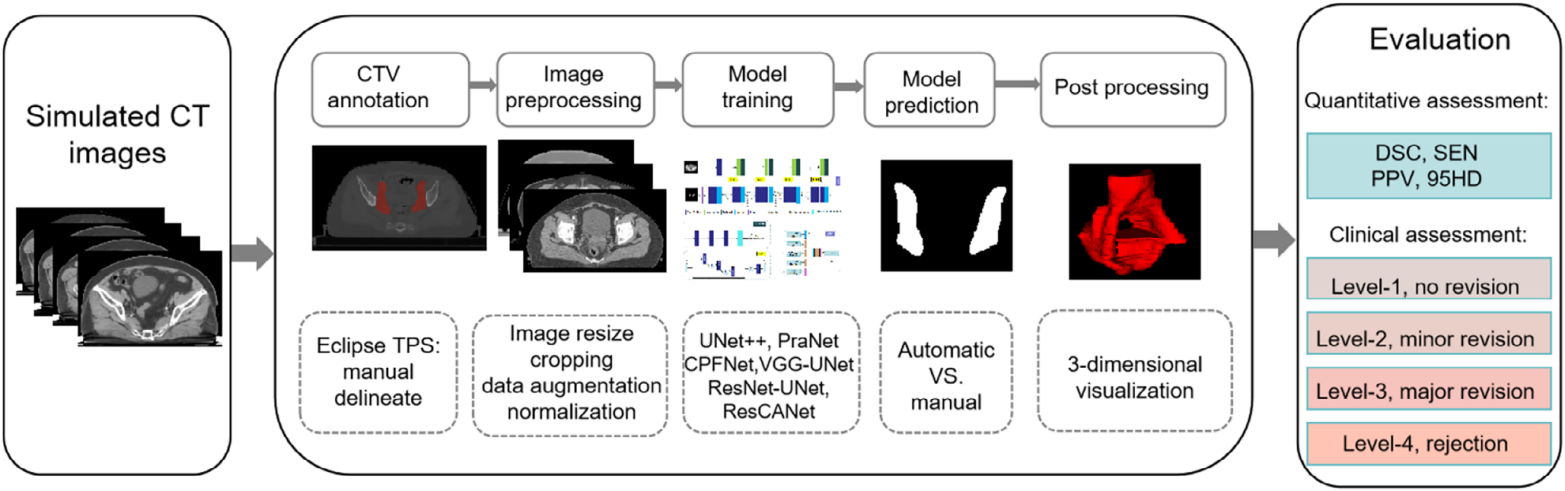
Overview of the workflow. The main processes include six components: clinical target volume (CTV) manual delineation in the treatment planning system (TPS), a preprocessing operations before model training, comparison of model prediction, and 3‐dimensional reconstruction. The evaluation includes quantitative geometric metrics and clinical assessment.

**FIGURE 3:**
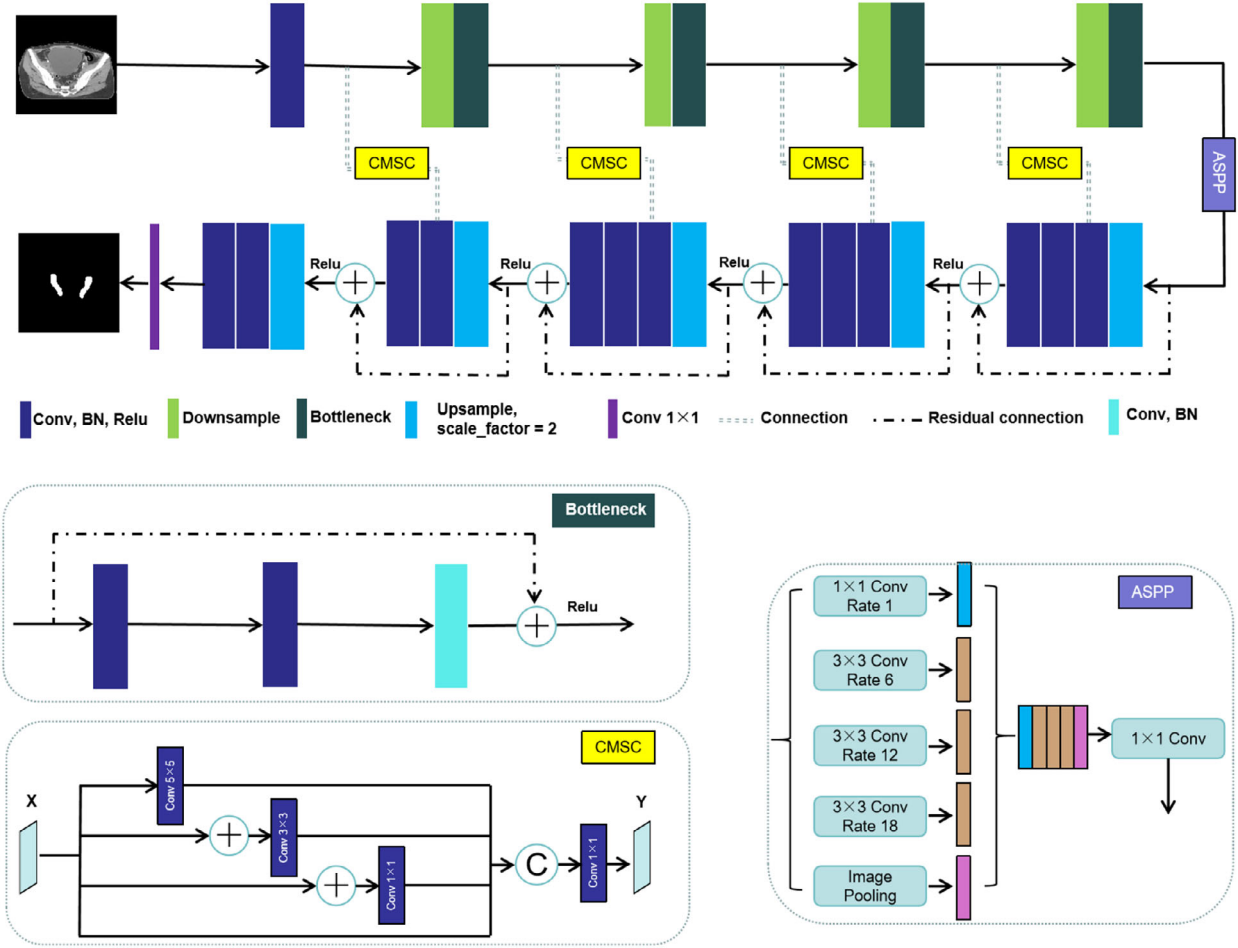
The proposed architecture of ResCANet.

An illustration of our research workflow is shown in Figure 2. The network was based on the open‐source library Pytorch 1.7.0 framework, Python 3.6.9, and the reference implementation of U‐Net.^14^ A new architecture called ResCANet, which is a typical U‐Net variant, is proposed and presented in Figure 3. The original feature extraction part was replaced with ResNet50^15^ as the backbone in encoder layers. The CT image features are extracted using the encoder with a residual structure. The decoding part is the bilinear interpolation (scale_factor = 2) successively restores the original dimension of the input data through the upsample block. The convolutional block output in the encoder is concatenated with the input to the corresponding one in the decoder. The upsampling layers were operated in a reverse way. Additionally, cascade multi‐scale convolution (CMSC) modules^16^ were added to the skip connections. The performances of U‐Net‐based segmentation models may be weakened if we concatenate these feature maps directly using skip connections. CMSC was used to eliminate semantic differences between different depth feature layers. The atrous spatial pyramid pooling (ASPP)^17^ was added in the deepest feature layer, which combines the semantic information of different receptive fields without losing information to improve the segmentation accuracy. The network was an end‐to‐end semantic segmentation framework that could achieve pixel‐wise labeling in CT images. Finally, a 1 × 1 convolution was used to convert the output channel to a number of classes to obtain the output of the 2D feature map.

### 2.3 Model 5‐fold cross‐training‐validation details

A total of 189 cervical cancer patients were used as the training set and 47 patients as the internal validation set. A 5‐fold cross‐validation strategy was used. The optimizer is Adam. The total loss function used in this study is a combination of the Dice loss function (Dice_Loss_) and Cross‐Entropy loss function (CE_Loss_), which is shown below. The detailed formula of Dice_Loss_ and CE_Loss_ is listed in reference.^18^

(1) $$\mathrm{Total}\mathrm{Loss}=0.75*\mathrm{Dic}e_{\mathrm{Loss}}+0.25*CE_{\mathrm{Loss}}$$

A cosine annealing scheduler was used (learning rate: 10^−4^, weight decay: 10^−4^). The training was terminated when the model loss did not decline in 100 epochs. The batch size was set as 28. Data augmentation techniques such as rotation, translation, flipping, and cropping were applied. The pre‐trained weights were obtained from the large‐scale trained on the ImageNet datasets.^19^ The networks were run on a server with an NVIDIA A100 graphics card and 32 GB RAM.

### 2.4 Quantitative geometric metrics evaluation

The automatic delineation performance was evaluated using the geometric metrics as follows, dice similarity coefficient (DSC), sensitivity (SEN), positive predictive value (PPV), and 95% Hausdorff distance (95HD), which were seen below.

(2) $$\mathrm{DSC}=\frac{2\times \mathrm{TP}}{\mathrm{FP}+2\mathrm{TP}+\mathrm{FN}}\times 100\%$$

(3) $$\mathrm{SEN}=\frac{\mathrm{TP}}{\mathrm{TP}+\mathrm{FN}}\times 100\%$$

(4) $$\mathrm{PPV}=\frac{\mathrm{TP}}{\mathrm{TP}+\mathrm{FP}}\times 100\%$$

where the TP represents the number of true positive pixels predicted to be positive pixels, FP represents the number of true negative pixels predicted to be positive pixels, and FN represents the number of true positive pixels predicted to be negative pixels. DSC reflected the spatial overlap degree used for segmentation performance evaluation. SEN is the proportion of TP number in the true pixels, while PPV is the proportion of TP number in the predicted pixels. HD represents the distance between the surface point sets of the calculated true pixels and the predicted pixels. 95HD represents the largest surface‐to‐surface distance among the closest 95% surface voxels.

(5) $$\mathrm{HD}\left(C,P\right)=\max\left(h\left(C,P\right),h\left(P,C\right)\right)$$

where h(C,P) = max{min||c‐p||}(c∈C, p∈P) and h(P,C) = max{min||p‐c||}(p∈P, c∈C).

Furthermore, our proposed model was compared with UNet++,^20^ PraNet,^21^ CPFNet,^22^ VGG‐UNet,^23^ and ResNet‐UNet^24^ in terms of automatic delineation of quantitative geometric metrics performance.

### 2.5 Oncologist clinical evaluation

The automatic delineation results in internal and external testing sets for cervical cancer and endometrial cancer were further evaluated visually by a senior oncologist in gynecologic oncology. In this work, the best‐predicted results obtained from our proposed model were analyzed for qualitative evaluation. In our radiotherapy center, the mean time of slice‐by slice delineation process for CTV takes more than 1 h to finish. We thus judged our prediction results in 4‐level criteria. Level‐1, no revision (delineation acceptable for therapy). Level‐2, minor revision (need for less than 30 min to revise the CTV). Level‐3, major revision (need for 30 to 60 min to revise the CTV), and Level‐4, rejection (need for more than 60 min to revise the CTV).

## 3 RESULTS

### 3.1 Performance of geometric metrics

We first quantified the geometric metrics results of different models, as shown in Table 1. ResCANet exhibited the best comprehensive performance, outperforming other networks on the majority of DSC, SEN, and 95HD metrics in internal and external cervical cancer and endometrial cancer testing, which demonstrated the superiority of our proposed network. The statistical analysis is also presented in Table 1. The five existing models were compared with the proposed ResCANet using paired *t*‐test by Python “scipy.stats” package. There were statistical differences (*P* < 0.05) in most evaluation metrics.

**TABLE 1:** Comparison of the automatic delineation performance in internal and external cervical cancer and endometrial cancer using 5‐fold cross‐validation (mean ± std).

Metrics	Type	UNet++	PraNet	CPFNet	VGG‐UNet	ResNet‐UNet	ResCANet (ours)
DSC(%)↑	CC1	74.5 ± 10.7 *P* = 0.473	73.9 ± 11.1 *P* = 0.105	74.5 ± 10.9 *P* = 0.620	74.7 ± 11.3 *P* = 0.842	74.6 ± 11.2 *P* = 0.704	**74.8 ± 11.0**
	CC2	71.0 ± 14.4 *P* = 0.001	70.5 ± 14.7 *P* < 0.001	73.1 ± 14.8 *P* = 0.676	72.2 ± 14.8 *P* = 0.074	68.4 ± 13.9 *P* < 0.001	**73.4 ± 14.6**
	EC	77.0 ± 5.3 *P* = 0.869	74.7 ± 5.9 *P* < 0.001	77.0 ± 5.3 *P* = 0.800	76.4 ± 5.2 *P* = 0.278	75.4 ± 5.6 *P* = 0.001	**77.1 ± 5.4**
SEN(%)↑	CC1	79.5 ± 12.2 *P* < 0.001	78.5 ± 12.7 *P* < 0.001	81.5 ± 11.0 *P* = 0.890	81.6 ± 11.0 *P* = 0.959	79.8 ± 13.7 *P* = 0.023	**81.5 ± 12.9**
	CC2	66.9 ± 15.6 *P* < 0.001	68.2 ± 16.5 *P* < 0.001	72.6 ± 16.0 *P* = 0.680	72.3 ± 16.4 *P* = 0.561	65.6 ± 16.0 *P* < 0.001	**72.9 ± 16.0**
	EC	79.6 ± 11.1 *P* = 0.025	74.7 ± 5.9 *P* < 0.001	**82.2 ± 10.1** *P* = 0.131	80.5 ± 10.4 *P* = 0.437	78.5 ± 12.1 *P* < 0.001	81.1 ± 11.1
PPV(%)↑	CC1	**74.6 ± 15.8** *P* = 0.007	74.4 ± 15.3 *P* = 0.142	72.5 ± 15.7 *P* = 0.018	72.7 ± 15.7 *P* = 0.276	74.0 ± 14.0 *P* = 0.231	73.5 ± 15.2
	CC2	**77.6 ± 16.8** *P* < 0.001	75.0 ± 16.5 *P* = 0.609	75.0 ± 16.3 *P* = 0.518	73.4 ± 16.2 *P* < 0.001	73.5 ± 15.8 *P* < 0.001	75.3 ± 16.3
	EC	**77.0 ± 10.9** *P* = 0.001	76.2 ± 11.6 *P* = 0.172	74.6 ± 11.0 *P* = 0.035	74.9 ± 11.3 *P* = 0.357	74.9 ± 10.0 *P* = 0.187	75.5 ± 10.9
95HD(mm)↓	CC1	11.2 ± 5.4 *P* = 0.005	11.2 ± 5.8 *P* = 0.002	11.5 ± 5.8 *P* = 0.006	11.3 ± 6.0 *P* = 0.052	11.5 ± 5.77 *P* < 0.001	**10.5 ± 5.7**
	CC2	13.2 ± 4.3 *P* = 0.034	13.7 ± 4.5 *P* < 0.001	12.8 ± 4.6 *P* = 0.388	13.7 ± 5.4 *P* = 0.014	14.8 ± 5.2 *P* < 0.001	**12.5 ± 4.5**
	EC	10.4 ± 3.5 *P* = 0.684	11.1 ± 3.8 *P* < 0.001	10.7 ± 3.6 *P* = 0.205	11.2 ± 3.6 *P* < 0.001	11.9 ± 3.9 *P* < 0.001	**10.3 ± 3.6**

*Note*: CC1, cervical cancer in internal validation. CC2, cervical cancer in external validation. EC, endometrial cancer validation. The best results are **in bold**. ↑ means the higher value of this metric is better, and ↓ means the lower value of this metric is better.

### 3.2 Ablation experiments

To investigate the effectiveness of our network. Ablation experiments were conducted. We constructed a ResNet‐UNet baseline model and then the new module, CMSC and ASPP were re‐assembled from the ResNet‐UNet baseline step by step and compared the performance quantitatively. Table 2 presents the comparison results of our method with different combinations of components in both internal and external cervical cancer datasets and endometrial cancer datasets experiments. CMSC module could improve by 3.6% and 1.3% on DSC in external cervical cancer datasets and endometrial cancer datasets, respectively. ASPP module could improve 1.4% and 0.4% on DSC in external cervical cancer datasets and endometrial cancer datasets, respectively. The most other metrics could be improved as the modules are added. These comparisons show that the CMSC and ASPP could enhance the model's ability.

**TABLE 2:** Ablation experimental results of ResCANet.

	Different modules			Metrics			
	Base	CMSC	ASPP	DSC (%)↑	SEN (%)↑	PPV (%)↑	95HD (mm)↓
CC1	√	–	–	74.6 ± 11.2	79.8 ± 13.7	74.0 ± 14.0	11.5 ± 5.77
	√	√	–	74.6 ± 10.8	79.3 ± 12.4	74.5 ± 15.3	11.6 ± 5.5
	√	√	√	74.8 ± 11.0	81.5 ± 12.9	73.5 ± 15.2	10.5 ± 5.7
CC2	√	–	–	68.4 ± 13.9	65.6 ± 16.0	73.5 ± 15.8	14.8 ± 5.2
	√	√	–	72.0 ± 14.9	67.8 ± 15.6	78.2 ± 16.3	13.7 ± 4.9
	√	√	√	73.4 ± 14.6	72.9 ± 16.0	75.3 ± 16.3	12.5 ± 4.5
EC	√	–	–	75.4 ± 5.6	78.5 ± 12.1	74.9 ± 10.0	11.9 ± 3.9
	√	√	–	76.7 ± 6.3	79.4 ± 12.3	76.5 ± 10.7	11.2 ± 3.7
	√	√	√	77.1 ± 5.4	81.1 ± 11.1	75.5 ± 10.9	10.3 ± 3.6

*Note*: CC1, cervical cancer in internal validation. CC2, cervical cancer in external validation. EC, endometrial cancer validation. ↑ means the higher value of this metric is better and ↓ means the lower value of this metric is better.

### 3.3 Automatic delineation effect

**FIGURE 4:**
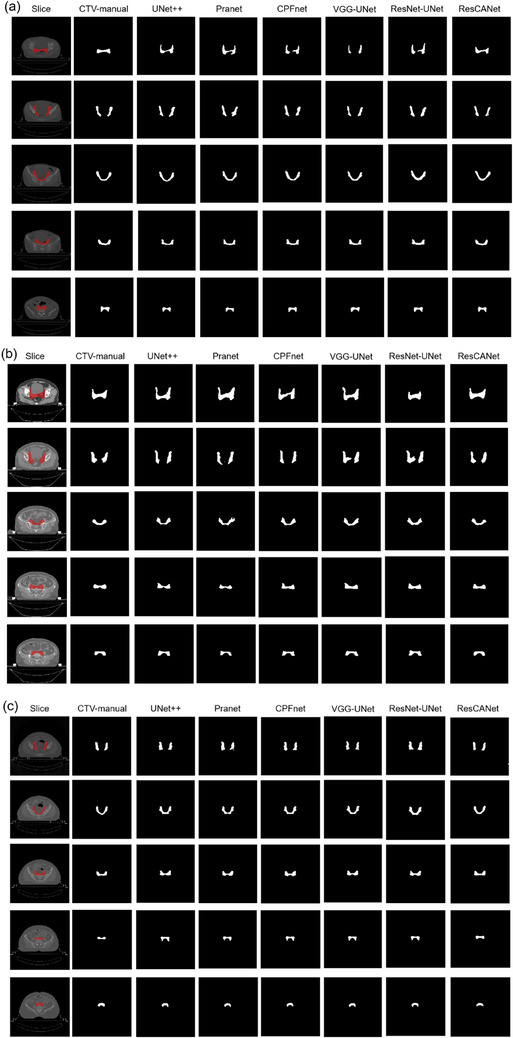
Comparison of delineation effect of different models. (a) Internal cervical cancer validation, (b) external cervical cancer validation, and (c) endometrial cancer validation.

Figure 4a–c shows the automatic delineation effect of internal and external cervical cancer and endometrial cancer, respectively. The first row shows the different simulated CT slices. The second row shows the corresponding manual delineation, and rows 3–8 show the automatic delineation results by different methods. ResCANet achieved the best automatic delineation effect in the models.

### 3.4 Clinician assessment

**FIGURE 5:**
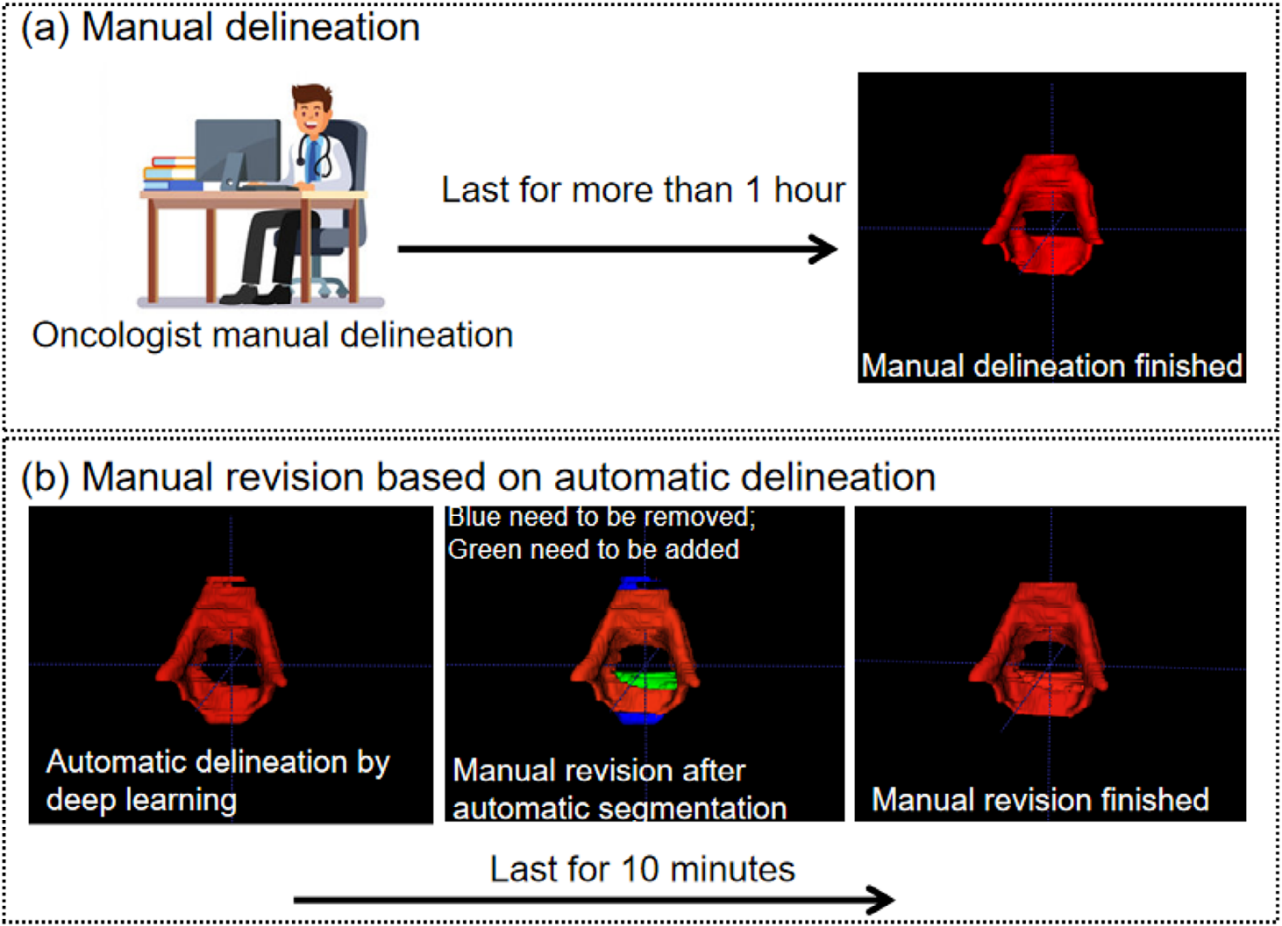
The 3‐dimensional visualization for revising CTV. (a) Manual delineation lasts for more than 1 h. (b) Manual revision based on automatic delineation (last for 10 min).

**FIGURE 6:**
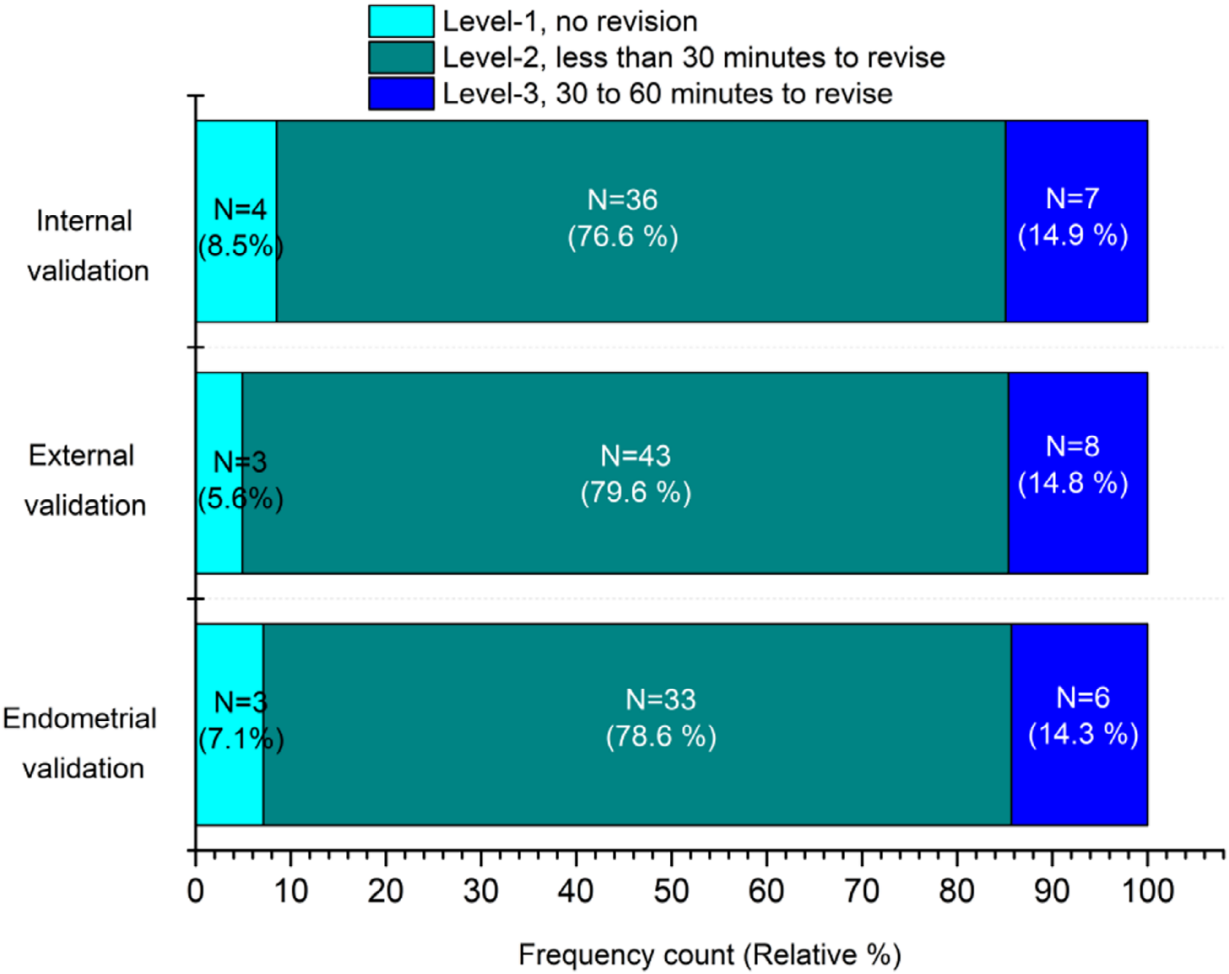
Frequency counts and relative (%) distribution of each level for cervical cancer and endometrial cancer testing in oncologist's scores.

The automatic delineation results by ResCANet of cervical cancer and endometrial cancer testing cases were reviewed slice‐by‐slice by a senior oncologist. Figure 5 presents an example of manual delineation and manual revision based on DL‐aided prediction. Although the parametrial area (Figure 5b) green part) could not be predicted accurately, the revision time was only 10 min in this example. Figure 6 shows the distribution of oncologist's scores on cervical cancer and endometrial cancer testing cases. In internal cervical cancer validation, four patients (8.5%) were Level‐1 (no revision). Thirty‐six patients (76.6%) were Level‐2 (minor revision). Seven patients (14.9%) were Level‐3 (major revision). In external cervical cancer validation, three patients (5.6%) were Level‐1 (no revision). Forty‐three patients (79.6%) were Level‐2 (minor revision). Eight patients (14.8%) were Level‐3 (major revision). In the endometrial cancer testing datasets, of the 42 patients’ CTV reviewed, three patients (7.1%) were Level‐1 (no revision). Thirty‐three patients (78.6%) were Level‐2 (minor revision). Six patients (14.3%) were Level‐3 (major revision).

## 4 DISCUSSION

CTV delineation is indispensable but a time‐consuming step for ensuring optimal radiotherapy planning in patients with cervical cancer. Accurate delineation affects the radiotherapy efficacy. Therefore, it is meaningful to develop an intelligent CTV delineation tool to assist clinicians in completing the delineation while saving medical resources. The cervical cancer datasets were trained in one center's datasets based on the developed network, and then the performance of the model was validated on internal and external cervical cancer and endometrial cancer cases. To the best of our knowledge, this is the first time we have studied the cervical cancer model and to tested the model in endometrial cancer cases to improve its applicability. Our results suggest that the proposed model is able to assist clinicians in rapidly delineating the CTV.

The shape of CTV is greatly affected by the surrounding organs such as the bladder and rectum, and the peripheral contour is not clear, or there is no obvious boundary, which makes it difficult to use DL to automatically delineate CTV. Previous studies are based on preoperative cervical cancer with a uterus^9^ or postoperative CTV delineation study without a uterus,^11^ but in a clinic setting, we need an effective tool to delineate CTV for cervical cancer in different tumor staging. This makes the segmentation task even more difficult. A novel DL model is therefore proposed to improve our delineation accuracy. The proposed ResCANet network combined the advantages of spatial channel dual attention and spatial pyramid pooling with existing ResNet‐UNet, and the CMSC was added to the skip connection. CMSC can integrate these multi‐scale feature maps together to bridge the semantic gap, so as to retain the feature information of small objects in the generated multi‐scale feature mapping. ASPP was added to acquire multi‐scale information, which was inspired by deeplabv3.^17^ The ablation experiments also proved the effectiveness of the added modules based on ResNet‐UNet. As shown in Table 1, ResCANet outperformed the other five models in the internal and external cervical cancer testing cases, especially DSC and 95HD, clearly illustrating the effectiveness of newly added modules for CTV delineation. Our model still has advantages in endometrial cancer testing cases. In this study, the DSC reached 0.756 and 0.766 in external cervical cancer and endometrial cancer validation, respectively. Similarly, the DSC/95HD of the pelvic lymph drainage area (dCTV1) and parametrial area (dCTV2) in the study by Ma et al.^12^ is 0.88/21.60 and 0.70/22.44 mm, respectively. Shi et al.^25^ reported a DSC of 0.688∼0.792 using different network structures for the cervical cancer CTV delineation. However, the DSC of our models is not as good as previous studies. In Ju et al.’s^26^ study, a DSC of 0.82 was achieved for the CTV delineation for cervical cancer radiotherapy. Liu et al.^9^ reported a DSC of 0.88 using a three‐stage multicenter randomized controlled evaluation for the CTV delineation for cervical cancer radiotherapy. Hou et al.^27^ attained the optimal DSCs for cervical_radical_ (0.85 ± 0.03), cervical_postoperative_ (0.85 ± 0.02) by the existing U‐Net and V‐Net. This indicates that there is still room for improvement in the performance of our model. Other factors that may affect the performance are the quality of the images and manual delineation quality.^11^ Wang et al.^28^ observed that DL based model generated structures with an average DSC of 0.77 for the CTV in the dependent testing case, which had comparability to our study. In addition, the generalization of our model was further validated in endometrial cancer cases, which showed a more robust performance in our network.

As shown in Figure 4, whether it is cervical cancer or endometrial cancer, ResCANet achieved better automatic delineation performance among the evaluated models in most of the slices. Additionally, in our study, we added oncologist clinical evaluation, which was not included in numerous studies.^11^, ^29^, ^30^, ^31^ As shown in Figure 5, the CTV was revised according to the predicted results. This artificial intelligence‐aided methods could improve the CTV delineation efficiency greatly. It is noteworthy that the quality scores were decided by calculating thresholds of DSC and HD or other geometric metrics in previous studies.^32^, ^33^, ^34^ In our study, we recorded the revision time on CTV, which was deemed more accurate because the cranial‐caudal boundary (Figure 5b) of the CTV could be erased through a 3‐dimensional brush tool in a very short time. We thought that the oncologist's scores were related to the complexity of the tumor that needed to be segmented. The quick DL‐aided revision can't be revealed by geometric metrics such as DSC. Furthermore, our results found that 85% of cases needed minor or no revision, which was similar to previous study.^27^

External validation between different centers and treatment sites could relieve the challenges associated with building a general DL model. The proposed network, which outperformed the existing models had investigated the problem of generalization of the CTV automatic delineation model to various radiotherapy institutions. The validation results showed the feasibility and reasonable accuracy in the CTV automatic delineation for both radical and postoperative cervical cancer cases between institutions. The proposed network can also be applied to endometrial cancer with a wider clinical application.

There are limitations in the present work. Firstly, there is still room for improvement in the accuracy of CTV automatic delineation. Secondly, a senior oncologist accomplished the clinical assessment, which may hinder the inter‐observer variations. Thirdly, more external validations are needed for further clinical applications.

## 5 CONCLUSIONS

We proposed a state‐of‐the‐art DL network, which had good generalization and robustness. The results demonstrate that automatic delineation achieved acceptable accuracy and shows the potential to improve clinical efficiency for radiotherapy of both cervical cancer and endometrial cancer.

## AUTHOR CONTRIBUTIONS

Zhe Wu performed the experiments and the statistical analysis. Dong Wang, Cheng Xu, Shengxian Peng, and Lihua Deng drafted the manuscript. Mujun Liu contributed to the design of the study. Yi Wu reviewed the manuscript and acquired the funding. All authors read and approved the final manuscript.

## CONFLICT OF INTEREST STATEMENT

The authors declare no conflicts of interest.

## References

[1] Vu M , Yu J , Awolude OA , Chuang L . Cervical cancer worldwide. Curr Probl Cancer. 2018;42:457‐465. 30064936 10.1016/j.currproblcancer.2018.06.003

[2] Gopalani SV , Janitz AE , Campbell JE . Trends in cervical cancer incidence and mortality in Oklahoma and the United States, 1999–2013. Cancer Epidemiol. 2018;56:140‐145. 30176544 10.1016/j.canep.2018.08.008 PMC6408322

[3] Rahimy E , von Eyben R , Lewis J , et al. Evaluating dosimetric parameters predictive of hematologic toxicity in cervical cancer patients undergoing definitive pelvic chemoradiotherapy. Strahlenther Onkol. 2022;198(9):773‐782. 35059758 10.1007/s00066-021-01885-z

[4] Court LE , Dong L , Taylor N , et al. Evaluation of a contour‐alignment technique for CT‐guided prostate radiotherapy: an intra‐ and interobserver study. Int J Radiat Oncol Biol Phys. 2004;59(2):412‐418. 15145157 10.1016/j.ijrobp.2003.10.023

[5] Zhou S , Nie D , Adeli E , et al. Semantic instance segmentation with discriminative deep supervision for medical images. Med Image Anal. 2022;82:102626. 36208573 10.1016/j.media.2022.102626

[6] Polat H . Multi‐task semantic segmentation of CT images for COVID‐19 infections using DeepLabV3+ based on dilated residual network. Phys Eng Sci Med. 2022;45(2):443‐455. 35286619 10.1007/s13246-022-01110-w PMC8919169

[7] Liu L , Wu FX , Wang YP , et al. Multi‐Receptive‐Field CNN for Semantic Segmentation of Medical Images. IEEE J Biomed Health Inform. 2020;24(11):3215‐3225. 32790636 10.1109/JBHI.2020.3016306

[8] Nie S , Wei Y , Zhao F , et al. A dual deep neural network for auto‐delineation in cervical cancer radiotherapy with clinical validation. Radiat Oncol. 2022;17(1):182. 36380378 10.1186/s13014-022-02157-5 PMC9667653

[9] Liu Z , Chen W , Guan H , et al. An adversarial deep‐learning‐based model for cervical cancer CTV segmentation with multicenter blinded randomized controlled validation. Front Oncol. 2021;11:702270. 34490103 10.3389/fonc.2021.702270 PMC8417437

[10] Kim N , Chang JS , Kim YB , et al. Atlas‐based auto‐segmentation for postoperative radiotherapy planning in endometrial and cervical cancers. Radiat Oncol. 2020;15(1):106. 32404123 10.1186/s13014-020-01562-y PMC7218589

[11] Xiao C , Jin J , Yi J , et al. RefineNet‐based 2D and 3D automatic segmentations for clinical target volume and organs at risks for patients with cervical cancer in postoperative radiotherapy. J Appl Clin Med Phys. 2022;23(7):e13631. 35533205 10.1002/acm2.13631 PMC9278674

[12] Ma CY , Zhou JY , Xu XT , et al. Deep learning‐based auto‐segmentation of clinical target volumes for radiotherapy treatment of cervical cancer. J Appl Clin Med Phys. 2022;23(2):e13470. 34807501 10.1002/acm2.13470 PMC8833283

[13] Hodneland E , Kaliyugarasan S , Wagner‐Larsen KS , et al. Fully automatic whole‐volume tumor segmentation in cervical cancer. Cancers (Basel). 2022;14(10):2372. 35625977 10.3390/cancers14102372 PMC9139985

[14] Ronneberger O , Fischer P , Brox T . U‐net: convolutional networks for biomedical image segmentation. Int Conf on Medical Image Computing and Computer‐Assisted Intervention. 2015:234‐241.

[15] He KM , Zhang XY , Ren SQ , et al. Deep Residual Learning for Image Recognition[C]//2016 IEEE Conference on Computer Vision and Pattern Recognition (CVPR). IEEE; 2016:770‐778.

[16] Wang B , Wang L , Chen JY , et al. w‐Net: Dual Supervised Medical Image Segmentation Model with Multi‐Dimensional Attention and Cascade Multi‐Scale Convolution. Arxiv preprint arXiv:2012.03674. 2020, https://arxiv.org/abs/2012.03674

[17] Chen LC , Papandreou G , Schroff F , et al. Rethinking Atrous Convolution for Semantic Image Segmentation. Arxiv preprint arXiv:1706.05587. 2017, https://arxiv.org/pdf/1706.05587

[18] Duprez D , Trauernicht C , Simonds H , et al. Self‐configuring nnU‐Net for automatic delineation of the organs at risk and target in high‐dose rate cervical brachytherapy, a low/middle‐income country's experience. J Appl Clin Med Phys. 2023;24(8):e13988. 37042449 10.1002/acm2.13988 PMC10402684

[19] Deng J , Dong W , Socher R , et al. 2009 IEEE conference on computer vision and pattern recognition. Ieee; 2009. Imagenet: A large‐scale hierarchical image database; pp. 248‐255.

[20] Zhou Z , Siddiquee M , Tajbakhsh N , et al. UNet++: redesigning skip connections to exploit multiscale features in image segmentation. IEEE Trans Med Imaging. 2020;39:1856‐1867. 31841402 10.1109/TMI.2019.2959609 PMC7357299

[21] Fan DP , Ji GP , Zhou T , et al. Pranet: parallel reverse attention network for polyp segmentation. Proceedings of International Conference on Medical Image Computing And Computer‐Assisted Intervention, (Cham). 2020. 263‐273.

[22] Feng S , Zhao H , Shi F , et al. CPFNet: context pyramid fusion network for medical image segmentation. IEEE Trans Med Imaging. 2020;39(10):3008‐3018. 32224453 10.1109/TMI.2020.2983721

[23] Krishnamoorthy S , Zhang Y , Kadry S , et al. Framework to segment and evaluate multiple sclerosis lesion in MRI slices using VGG‐UNet. Comput Intell Neurosci. 2022;2022:4928096. 35694573 10.1155/2022/4928096 PMC9184172

[24] de Groof AJ , Struyvenberg MR , van der Putten J , et al. Deep‐learning system detects neoplasia in patients with Barrett's Esophagus with higher accuracy than endoscopists in a multistep training and validation study with benchmarking. Gastroenterology. 2020;158(4):915‐929.e4. 31759929 10.1053/j.gastro.2019.11.030

[25] Shi J , Ding X , Liu X , et al. Automatic clinical target volume delineation for cervical cancer in CT images using deep learning. Med Phys. 2021;48(7):3968‐3981. 33905545 10.1002/mp.14898

[26] Ju Z , Guo W , Gu S , et al. CT based automatic clinical target volume delineation using a dense‐fully connected convolution network for cervical cancer radiation therapy. BMC Cancer. 2021;21(1):243. 33685404 10.1186/s12885-020-07595-6 PMC7938586

[27] Hou Z , Gao S , Liu J , et al. Clinical evaluation of deep learning‐based automatic clinical target volume segmentation: a single‐institution multi‐site tumor experience. Radiol Med. 2023;128(10):1250‐1261. 37597126 10.1007/s11547-023-01690-x

[28] Wang J , Chen Y , Xie H , et al. Evaluation of auto‐segmentation for EBRT planning structures using deep learning‐based workflow on cervical cancer. Sci Rep. 2022;12(1):13650. 10.1038/s41598-022-18084-0 PMC9372087 35953516

[29] Ma CY , Zhou JY , Xu XT , et al. Clinical evaluation of deep learning‐based clinical target volume three‐channel auto‐segmentation algorithm for adaptive radiotherapy in cervical cancer. BMC Med Imaging. 2022;22(1):123. 35810273 10.1186/s12880-022-00851-0 PMC9271246

[30] Tian M , Wang H , Liu X , et al. Delineation of clinical target volume and organs at risk in cervical cancer radiotherapy by deep learning networks. Med Phys. 2023;50(10):6354‐6365. 37246619 10.1002/mp.16468

[31] Wong J , Huang V , Wells D , et al. Implementation of deep learning‐based auto‐segmentation for radiotherapy planning structures: a workflow study at two cancer centers. Radiat Oncol. 2021;16(1):101. 34103062 10.1186/s13014-021-01831-4 PMC8186196

[32] Duan J , Bernard ME , Castle JR , et al. Contouring quality assurance methodology based on multiple geometric features against deep learning auto‐segmentation. Med Phys. 2023;50(5):2715‐2732. 36788735 10.1002/mp.16299 PMC10175153

[33] Men K , Geng H , Biswas T , et al. Automated quality assurance of OAR contouring for lung cancer based on segmentation with deep active learning. Front Oncol. 2020;10:986. 32719742 10.3389/fonc.2020.00986 PMC7350536

[34] Chen X , Men K , Chen B , et al. CNN‐based quality assurance for automatic segmentation of breast cancer in radiotherapy. Front Oncol. 2020;10:524. 32426272 10.3389/fonc.2020.00524 PMC7212344

